# ‘Sweet poison’ and ‘mild medicine’: Different effects of collective narcissism and collective self‐esteem on ingroup versus outgroup conspiracy beliefs

**DOI:** 10.1111/bjop.70032

**Published:** 2025-09-26

**Authors:** Jia‐Yan Mao, Cai‐Yu Tian, Shen‐Long Yang, Jan‐Willem van Prooijen

**Affiliations:** ^1^ Department of Experimental and Applied Psychology VU Amsterdam Amsterdam The Netherlands; ^2^ School of Psychology University of Kent Canterbury UK; ^3^ Institute of Social Psychology, School of Humanities and Social Science Xi'an Jiaotong University Xi'an China; ^4^ The Netherlands Institute for the Study of Crime and Law Enforcement (NSCR) Amsterdam The Netherlands; ^5^ Department of Criminal Law and Criminology Maastricht University Maastricht The Netherlands

**Keywords:** collective narcissism, collective self‐esteem, conspiracy belief, instrumental treatment, perceived threat, system‐justifying belief, victim consciousness

## Abstract

Collective narcissism and non‐narcissistic ingroup positivity (notably collective self‐esteem) are associated differently with conspiracy beliefs. We conducted three cross‐sectional surveys in China and the United States that distinguished between ingroup and outgroup conspiracy beliefs, to explore the intricate relationships and underlying mechanisms of these variables. Studies 1 (*N* = 800) and 2 (*N* = 385) showed that, in China, collective narcissism was positively associated with outgroup conspiracy belief (partially mediated by increased perceived threat from the outgroup) and with ingroup conspiracy belief (partially mediated by increased instrumental treatment of ingroup members); collective self‐esteem was positively associated with outgroup conspiracy belief (fully mediated by increased victim consciousness), but negatively with ingroup conspiracy belief (fully mediated by increased system‐justifying belief). Study 3 (*N* = 397) only replicated the significant positive relationship between collective narcissism and outgroup conspiracy belief in a US sample, and the partial mediating effect of increased perceived threat from the outgroup in it, while the other three paths were not statistically significant. These findings suggest that the association between different forms of ingroup positivity (narcissistic versus non‐narcissistic) and conspiracy beliefs is influenced both by the identity of the conspirators (ingroup versus outgroup) and cultural context.

## BACKGROUND

In January 2021, supporters of President Donald Trump rioted at the US Capitol, claiming their actions were patriotic while adhering to election fraud conspiracy theories (Zhai & Yan, 2023). Support for the attacks, and for Donald Trump's use of force to stay in power, was associated with collective narcissism (Keenan & Golec de Zavala, 2021). This example underscores that not all ingroup positivity has positive social implications. Previous researchers have developed two concepts of ingroup positivity. Collective narcissism is a defensive form, characterized by a belief that the ingroup is superior and deserving of privileges, coupled with a suspicion that the outside world is not giving the ingroup the recognition and preferential treatment it deserves (de Golec Zavala et al., 2009; Golec de Zavala & Keenan, 2021). The other is non‐narcissistic ingroup positivity such as collective self‐esteem, meaning the extent to which individuals confidently hold positive evaluations of their ingroup independent of others' perceptions of the ingroup (Cichocka, 2016).

Since collective narcissism and collective self‐esteem both involve positive feelings for the ingroup, there is some empirical and conceptual overlap (Golec de Zavala et al., 2013; Marchlewska et al., 2020). Once this overlap is accounted for, however, psychological and behavioural correlates between the two tend to diverge (Sternisko et al., 2023). Ingroup positivity that does not contain narcissistic, defensive components (e.g. collective self‐esteem) is associated with constructive ingroup and intergroup attitudes and behaviours, such as higher ingroup loyalty, a greater willingness to benefit the ingroup (Cichocka, 2016; Marchlewska et al., 2020) and a higher willingness to collaborate in prevention behaviours to combat the COVID‐19 pandemic (Chan et al., 2021; Federico et al., 2021). Collective narcissism predicts more damaging attitudes and behaviours, such as hostile and aggressive attitudes toward outgroups, oversensitivity to threats and criticism (Cislak et al., 2021; Lyons et al., 2010; Wang et al., 2021) and belief in outgroup‐related conspiracy theories (Golec de Zavala et al., 2022).

Previous research has suggested that ingroup positivity can motivate conspiracy beliefs, which are defined as explanatory beliefs that attribute significant political or social events to a group or individuals who colluded in secret to achieve malevolent goals (Goertzel, 1994; Green & Douglas, 2018). This is because conspiracy theories help people develop, maintain and protect a positive social identity (van Prooijen, 2024). However, several studies have found different relationships between collective narcissism, collective self‐esteem and conspiracy beliefs (e.g. Biddlestone et al., 2022; Cichocka, Marchlewska, Golec de Zavala, & Olechowski, 2016; Golec de Zavala et al., 2022; Wang et al., 2021). We propose that these specific relationships with conspiracy beliefs may depend on the target of the conspiracy accusations (Golec de Zavala et al., 2022).

A common distinction is between ‘ingroup’ versus ‘outgroup’ conspiracy theories (e.g. Mao, Van Prooijen, et al., 2024), referring to whether the conspirators are from within or outside a valued community. Specifically, people may perceive conspiracies within their own community such as a national government, their own political party or their own religious community (defined as ingroup conspiracy theories); or people may perceive conspiracies outside of their community such as a foreign government, a rival political party or other religious communities (defined as outgroup conspiracy theories). People have different motivations for supporting ingroup and outgroup conspiracy theories (Bertin et al., 2022; Mao et al., 2025; van Prooijen & van Lange, 2014). Therefore, identifying the underlying factors associated with ingroup and outgroup conspiracy theories (e.g. collective narcissism, collective self‐esteem) will help shed light on the motivational basis for the formation of conspiracy theories (Biddlestone et al., 2022).

In addition to the target of the conspiracy accusations, cultural background is also likely to be an important factor affecting these relationships. In the current research, we aim to explore the relationships between collective narcissism, collective self‐esteem and intergroup conspiracy theories in China (Studies 1 and 2) and the United States (Study 3). These two countries were selected based on the following considerations: (a) Previous inconsistent findings were mainly from these two countries (e.g. Cichocka, Marchlewska, Golec de Zavala, & Olechowski, 2016; Zhai & Yan, 2023); (b) previous studies have underscored that citizens of these two countries perceive each other as rival outgroups (e.g. Mao, Zeng, et al., 2024; van Prooijen & Song, 2021); (c) Focusing on these two countries helps assess the differences among these effects.

### Collective narcissism and conspiracy theories

In a meta‐analysis, Golec de Zavala et al. (2022) found a robust association between collective narcissism and outgroup conspiracy beliefs. For example, collective narcissism of Poles predicts an endorsement of conspiratorial stereotypes about Jews (Golec de Zavala & Cichocka, 2012) and belief in the Smolensk conspiracy theory blaming Russia for the plane crash (Cichocka, Marchlewska, Golec de Zavala, & Olechowski, 2016). Similarly, Americans' collective narcissism foreshadows an endorsement of conspiracy theories involving foreign governments (Cichocka, Marchlewska, Golec de Zavala, & Olechowski, 2016; van Prooijen & Song, 2021). Chinese collective narcissism is also linked to belief in conspiracy theories about the United States (e.g. Mao, Zeng, et al., 2024; Wang et al., 2021; Zhai & Yan, 2023). Moreover, collective narcissism of Catholicism predicts conspiracy theories that gender studies and gender equality activists, who were regarded as a religious outgroup, secretly plotted to undermine traditional values and social arrangements (Marchlewska et al., 2019).

The link between collective narcissism and outgroup conspiracy theories can be explained by the intergroup threat sensitivity of collective narcissists (Bertin et al., 2022; Cichocka, Marchlewska, & Biddlestone, 2022; Cichocka, Marchlewska, Golec de Zavala, & Olechowski, 2016). Specifically, collective narcissists are relatively sensitive to outgroup threats and tend to exaggerate them (Biddlestone et al., 2022; de Golec Zavala et al., 2009; Sternisko et al., 2023). Moreover, collective narcissists respond to this threat by disparaging or attacking the outgroup (de Golec Zavala et al., 2013; Lyons et al., 2010). Attributing malicious conspiracies to specific outgroups hence can be a means of preserving the group image, by offering a flattering explanation for why others do not acknowledge the greatness of the ingroup (Golec de Zavala et al., 2022). Previous research has also demonstrated that, after controlling for the overlap with non‐narcissistic ingroup positivity, perceived outgroup threat mediates the relationship between collective narcissism and outgroup conspiracy beliefs (e.g. Cichocka, Marchlewska, Golec de Zavala, & Olechowski, 2016). We therefore propose Hypothesis 1.

Hypothesis 1 Collective narcissism is positively associated with outgroup conspiracy beliefs—an effect that is mediated by perceived outgroup threat.

However, cumulative evidence finds that collective narcissism also predicts ingroup conspiracy beliefs (e.g. Biddlestone et al., 2022; Golec de Zavala & Federico, 2018). For example, in Poland, the United States and the United Kingdom, collective narcissism predicted stronger intentions to conspire against the ingroup and stronger belief in ingroup conspiracy theories (e.g. about one's government or work team; Biddlestone et al., 2022). Meta‐analyses show that collective narcissism correlates positively and significantly with various forms of individual narcissism, especially vulnerable narcissism (Golec de Zavala et al., 2019). For collective narcissists, self‐interest is more important than the ingroup image and the well‐being of its members (Cichocka & Cislak, 2020; Cislak et al., 2021; Golec de Zavala & Keenan, 2021). Due to a relative lack of empathy (Gronfeldt et al., 2023) and solidarity (Federico et al., 2021) with ingroup members, collective narcissists therefore tend to treat ingroup members instrumentally to achieve their own goals (Cichocka, Cislak, et al., 2022; LaCroix & Pratto, 2015). This process of treating ingroup members as means to an end for personal gain can be defined as instrumental treatment, as an operational definition of objectification (Cichocka, Cislak, et al., 2022; Gruenfeld et al., 2008).

As collective narcissists have a higher willingness to participate in conspiracies against the ingroup, they are likely to project their selfish motivations onto other ingroup members. Ingroup members thus become potential targets for conspiracy theories, and thus, this process is accompanied by increased ingroup conspiracy beliefs (Biddlestone et al., 2022; Douglas & Sutton, 2011). Instrumental treatment of ingroup members seems to be a way for collective narcissists to ‘exonerate’ themselves and plays an important role in the formation of their ingroup conspiracy beliefs. Therefore, we propose Hypothesis 2.

Hypothesis 2 Collective narcissism is positively associated with ingroup conspiracy beliefs—an effect that is mediated by instrumental treatment of ingroup members.

In general, these arguments suggest that collective narcissism is like a ‘sweet poison’ to the ingroup, hiding suspicion and threat to the ingroup under the cloak of exaggerated positivity. By contrast, collective self‐esteem, as a secure form of ingroup positivity, acts as a ‘mild medicine’ that predicts a more open and trusting attitude toward outgroup members (Golec de Zavala et al., 2013), which suppresses both outgroup and ingroup conspiracy theories (Cichocka, Marchlewska, Golec de Zavala, & Olechowski, 2016).

### Collective self‐esteem and conspiracy theories

According to the meta‐analysis by Golec de Zavala et al. (2022), non‐narcissistic ingroup positivity, such as collective self‐esteem (Luhtanen & Crocker, 1992) or positive ingroup regard (Brewer, 2001), was not significantly associated with belief in conspiracy theories. Unlike collective narcissism, however, there is relatively limited research on the relationship between collective self‐esteem (or other conceptualizations of non‐narcissistic ingroup positivity) and ingroup and outgroup conspiracy theories, and there are inconsistencies in these findings.

In comparison with collective narcissism, collective self‐esteem postulates a more secure perception of the ingroup. This positive yet secure ingroup positivity predicts a more open and trusting attitude toward outgroup members (Golec de Zavala et al., 2013), as well as a lower sensitivity to outgroup threats (Lyons et al., 2013), and thus may reduce suspicions of outgroup conspiracies (Cichocka, Marchlewska, Golec de Zavala, & Olechowski, 2016). Previous studies have found that after controlling for collective narcissism, collective self‐esteem (Luhtanen & Crocker, 1992) and other forms of non‐narcissistic ingroup positivity including ingroup identification (Cameron, 2004) and group‐level self‐investment (Leach et al., 2008), are negatively correlated with outgroup conspiracy beliefs (Cichocka, Marchlewska, Golec de Zavala, & Olechowski, 2016).

For the relationship between collective self‐esteem and ingroup conspiracy theories, non‐narcissistic ingroup positivity includes satisfaction with ingroup members, emotional attachment to other ingroup members and the importance of the ingroup to the self (Cichocka, Marchlewska, Golec de Zavala, & Olechowski, 2016). Moreover, non‐narcissistic ingroup positivity (e.g. national identification) negatively predicts the tendency to blame the ingroup for major social problems (Rotella & Richeson, 2013; Wang et al., 2021), which correlates with lower ingroup conspiracy beliefs (Mao, Zeng, et al., 2024). Previous studies have indeed found that after controlling for collective narcissism, group‐level self‐investment has a weak but significant negative correlation with ingroup conspiracy beliefs in the United States (e.g. Biddlestone et al., 2022; Cichocka, Marchlewska, Golec de Zavala, & Olechowski, 2016).

In China, however, patriotism, another form of non‐narcissistic ingroup positivity, has been found to have no significant relationship with either ingroup or outgroup conspiracy beliefs (Zhai & Yan, 2023). We believe this is because Zhai and Yan's study (Zhai & Yan, 2023) was conducted during the Covid‐19 pandemic, and the relationships between variables were likely influenced by the complex social context at the time. More generally, however, it might be speculated that, due to a unique history of victimization, victimhood has become part of Chinese identity, amplifying the link between collective self‐esteem and outgroup conspiracy belief. Furthermore, due to the one‐party system, Chinese people tend to strongly defend the system, thereby strengthening the negative correlation between collective self‐esteem and ingroup conspiracy belief. Given the implications of the meta‐analysis results, the limited evidence from previous studies and the inconsistencies in the findings from China, we do not formulate hypotheses about the relationships between collective self‐esteem and ingroup and outgroup conspiracy beliefs in Study 1, an exploratory study conducted in China.

### Current research overview

In the current research, we report three cross‐sectional survey studies that explore the complex relationships between collective narcissism, collective self‐esteem, and ingroup and outgroup conspiracy beliefs, as well as potential mediating mechanisms underlying each different path. Study 1 is an exploratory study to test the correlations between these variables in a Chinese context. Based on the findings of Study 1, Study 2 further tested Hypotheses 1, 2 and additional hypotheses about mediating processes in the Chinese context. Specifically, Study 2 tested the mediating role of perceived outgroup threat between collective narcissism and outgroup conspiracy beliefs, and the mediating role of instrumental treatment of ingroup members between collective narcissism and ingroup conspiracy beliefs. Study 2 also tested the links between collective self‐esteem and ingroup and outgroup conspiracy beliefs, along with possible mediating processes. Study 3 tested these hypotheses in an American context using the same measures as Study 2. Studies 1 and 2 were conducted with Chinese samples and Study 3 with an American sample.

### Open practices statement

All data and materials of the studies reported here are publicly available on the Open Science Framework (https://osf.io/a69se/files/osfstorage). For all the studies, we report all the measures (either in the method sections or the Supporting Information); data exclusions (if any) are reported in the method sections of the respective studies. All the studies reported here have formal ethical approval, and Studies 2 and 3 were preregistered (https://osf.io/wn27j).

## STUDY 1

Study 1 was a questionnaire survey with a large sample in mainland China.^1^ All participants completed measures of collective narcissism, collective self‐esteem, ingroup conspiracy beliefs and outgroup (the United States) conspiracy beliefs. Our purpose was to examine the complex relationships between different forms of ingroup positivity and intergroup conspiracy beliefs in a Chinese context.

### Method

#### Participants and design

Through the Credamo platform, a Chinese crowdsourcing site similar to Amazon's Mechanical Turk, we sent out a battery of questionnaires. 814 adult Chinese participants provided written informed consent, among which fourteen people were excluded due to a wrong answer on the attention test (e.g. ‘Please choose “neutral” when you see this question’). The final sample for data analysis consisted of 800 participants (313 male, 487 female), ranging in age from 19 to 71 years (*M*_age_ = 32.97, *SD* = 7.02).

#### Materials and procedure

We used the shorter version of the scale developed by de Golec Zavala et al. (2009) to measure collective narcissism, which consisted of five items (e.g. ‘If China had a major say in the world, the world would be a much better place’; 1 = *strongly disagree*, 7 = *strongly agree*, *α* = .59).

Collective self‐esteem was measured by using the scale developed by Luhtanen and Crocker (1992). The measurement consisted of sixteen items (e.g. ‘I am a worthy member of the social groups I belong to’; 1 = *strongly disagree*, 7 = *strongly agree*, *α* = .83). The question stem emphasized that participants should respond based on their feelings as members of the Chinese community.

Outgroup (American) conspiracy beliefs were assessed by adapting the measure of van Prooijen and Song (2021), which consisted of seven items (e.g. ‘The secret agency of the United States has been trying to influence political decision‐making in China’; 1 = *strongly disagree*, 7 = *strongly agree*, *α* = .92).

We adapted five items from the conspiracy mentality scale (Imhoff & Bruder, 2014) and compiled one additional item to measure ingroup conspiracy beliefs. We specifically adapted these items by specifying that ‘those at the top’ refer to the Chinese society and government (ingroup). An example item is ‘Most people do not recognize to what extent our life is determined by conspiracies secretly orchestrated by those at the top’ (1 = *strongly disagree*, 7 = *strongly agree*). The final measurement consisted of six items (*α* = .80).

The four variables above were measured using validated Chinese versions of the original scales, which we adopted or adapted as appropriate.^2^ In addition, we collected basic demographic information of the participants and paid each a small sum of money.

### Results

Descriptive statistics and correlational analyses of all variables are presented in Table 1. As shown in Table 1, collective narcissism is significantly positively correlated with collective self‐esteem and outgroup conspiracy beliefs, but not with ingroup conspiracy beliefs. Collective self‐esteem is correlated positively with outgroup conspiracy beliefs but negatively with ingroup conspiracy beliefs. In addition, there is no significant correlation between ingroup and outgroup conspiracy beliefs.

**TABLE 1:** Descriptive analysis and correlations (Study 1).

	*M*	*SD*	1	2	3	4
1. Collective narcissism	5.59	0.71	1	[0.21, 0.34]	[0.35, 0.46]	[−0.07, 0.07]
2. Collective self‐esteem	5.99	0.49	.28***	1	[0.12, 0.25]	[−0.43, −0.31]
3. Outgroup conspiracy belief	5.63	1.10	.41***	.19***	1	[−0.11, 0.02]
4. Ingroup conspiracy belief	3.18	1.16	.00	−.37***	−.05	1

*Note*: Below the diagonal are Pearson correlation coefficients; above the diagonal are the corresponding 95% confidence intervals.

*** *p* < .001.

**FIGURE 1:**
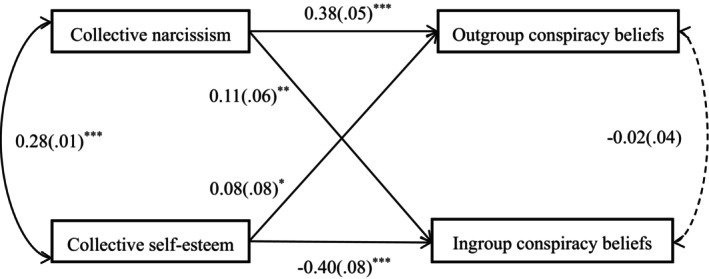
Effects of collective narcissism and collective self‐esteem on different types of conspiracy beliefs. *p < .05, **p < .01, ***p < .001. Entries are standardized regression coefficients with standard errors in parentheses. Dotted line indicates a non‐significant path.

However, given the empirical overlap between collective narcissism and collective self‐esteem, we adopt a path model to conduct a fully exploratory analysis. To save degrees of freedom, this analysis did not include control variables. The sample size meets the requirements for structural equation modelling of five to six participants per estimated parameter (Bentler & Chou, 1987). The analysis was run in the *lavaan* package of *R* (Rosseel, 2012). Results are displayed in Figure 1.

As the model is saturated, all fit indices indicated a perfect fit. We therefore focus our interpretation only on the individual paths. As can be discerned from Figure 1, collective narcissism was positively related to outgroup conspiracy beliefs, *β* = 0.384, *SE* = .051, *p* < .001, 95% CI [0.487, 0.689], and ingroup conspiracy beliefs, *β* = 0.110, *SE* = .055, *p* = .001, 95% CI [0.071, 0.287]. Collective self‐esteem was positively related to outgroup conspiracy beliefs, *β* = 0.081, *SE* = .075, *p* = .015, 95% CI [0.035, 0.327], but negatively related to ingroup conspiracy beliefs, *β* = −0.400, *SE* = .080, *p* < .001, 95% CI [−1.099, −0.786]. When gender and age were included as covariates, all the paths were still significant and in the same direction.

### Discussion

The results of Study 1 indicate that in China, after controlling for the overlap between collective narcissism and collective self‐esteem, there is a significantly positive relationship between collective narcissism and both ingroup and outgroup conspiracy beliefs. Moreover, collective self‐esteem is positively associated with outgroup conspiracy beliefs but negatively with ingroup conspiracy beliefs. These findings are consistent with Hypotheses 1 and 2, although the questionnaire did not include the hypothesized mediating variables. In Study 2, we will further test the relationships between collective self‐esteem and conspiracy beliefs in China, while also examining the hypothesized mediating processes.

## STUDY 2

Based on the positive correlation between collective self‐esteem and outgroup conspiracy beliefs found in Study 1, we suggest that this is likely mediated by victim consciousness. Throughout modern history, China has been invaded and isolated by external forces (including the United States), and victim consciousness (defined as the belief that one's ingroup is the victim of constant persecution by particular enemies) hence may have become part of the national psyche (Li et al., 2023; Vollhardt & Bilali, 2015). Meanwhile, many current intergroup interactions (e.g. the US–China Trade War) have reinforced Chinese victim consciousness (Wang et al., 2021). We speculate that the positive ingroup identity in collective self‐esteem motivates Chinese to identify with the ingroup's victim identity, potentially stimulating victim consciousness. In addition, victim consciousness is also linked to outgroup conspiracy beliefs (Bilewicz, 2022; Pantazi et al., 2022; Wang, 2014). Therefore, we propose Hypothesis 3a.

Hypothesis 3a In China, after controlling for collective narcissism, collective self‐esteem is positively associated with outgroup conspiracy beliefs—an effect that is mediated by victim consciousness.

We further propose that the negative correlation between collective self‐esteem and ingroup conspiracy beliefs may be mediated by system‐justifying belief. Previous research has found that in China, collective self‐esteem negatively predicts the tendency to ascribe blame to the ingroup, which in turn predicts denialist conspiracy beliefs (e.g. COVID‐19 is a hoax). The logic behind this process is that ‘it's our doing, so it's not so bad’ (Wang et al., 2021). Put differently, collective self‐esteem reflects a motivation to justify, legitimize and absolve the ingroup. This is close to the connotation of system‐justifying belief, which can be defined as the belief that the social system and the status quo are legitimate (Kay & Jost, 2003). Additionally, Mao, Zeng, et al. (2024) have demonstrated a significant negative correlation between system‐justifying belief and ingroup conspiracy beliefs in the Chinese context. We therefore propose Hypothesis 4a.

Hypothesis 4a In China, after controlling for collective narcissism, collective self‐esteem is negatively associated with ingroup conspiracy beliefs—an effect that is mediated by system‐justifying belief.

Similar to Study 1, Study 2 was a questionnaire survey conducted in mainland China. In addition to the measures of the variables in Study 1, we also included measures of the possible mediating variables outgroup threat, instrumental treatment of ingroup members, victim consciousness and system‐justifying belief. We aimed to test Hypotheses 1, 2, 3a and 4a.

### Method

#### Participants and design

We recruited 402 participants from the Tencent Questionnaire platform, another Chinese crowdsourcing site similar to Credamo. Seventeen participants were excluded for failing our attention test (as in Study 1). The final sample for data analysis consisted of 385 participants (137 male, 248 female), ranging in age from 18 to 64 years, with a mean age of 26.25 years (*SD* = 7.15).

#### Materials and procedure

Since the 5‐item scale's reliability was suboptimal in Study 1, we used the original version of the scale developed by de Golec Zavala et al. (2009) to measure collective narcissism, which consisted of nine items (e.g. ‘I wish other groups would more quickly recognize the authority of China’; 1 = *strongly disagree*, 7 = *strongly agree*, *α* = .75).

The 16‐item collective self‐esteem scale (*α* = .85) and 7‐item outgroup (American) conspiracy beliefs scale (*α* = .93) were measured as in Study 1. We added another self‐compiled item (i.e. ‘Whenever there is a major social event, those at the top will not hide information from the public’) to the 6‐item ingroup conspiracy beliefs scale of Study 1, resulting in a new measure of ingroup conspiracy beliefs with seven items (*α* = .66).

We adapted the objectification scale developed by Gruenfeld et al. (2008) to measure instrumental treatment of ingroup members. Participants were instructed to respond to these items by first recalling their own experiences of interacting or working with Chinese government officials or government workers in their social lives, and then evaluating their own relationships with these government officials or government workers based on their true feelings. The measurement consisted of seven items (e.g. ‘I think more about what they can do for me than what I can do for them’; 1 = *strongly disagree*, 7 = *strongly agree*, *α* = .65).

We adapted the measurement of outgroup threat by de Golec Zavala et al. (2009) to assess Chinese participants' perceived threat from America. The measurement consisted of four items (e.g. ‘America's nuclear weapons are a critical threat to China’; 1 = *strongly disagree*, 7 = *strongly agree*, *α* = .93).

We used the scale developed by Szabó and Csertő (2023) to measure victim consciousness, which consisted of eight items (e.g. ‘No other country went through similar hardships as China’; 1 = *strongly disagree*, 7 = *strongly agree*, *α* = .88).

We used the scale developed by Kay and Jost (2003) to measure system‐justifying belief, which consisted of eight items (e.g. ‘In general, I find Chinese society to be fair’; 1 = *strongly disagree*, 7 = *strongly agree*, *α* = .87).

For collective narcissism and system‐justifying belief, we used validated Chinese versions of the scales. For instrumental treatment of ingroup members, outgroup threat and victim consciousness, we provided the first Chinese translations. The translation procedures for these scales are detailed in the online Supporting Information. Basic demographic information was also collected, and all participants received a small monetary reward.

## RESULTS

Descriptive statistics and correlational analyses of all variables are presented in Table 2. The correlations between collective narcissism, collective self‐esteem, and ingroup and outgroup conspiracy beliefs are consistent with the findings in Study 1.

**TABLE 2:** Descriptive analysis and correlations (Study 2).

	*M*	*SD*	1	2	3	4	5	6	7	8
1. Collective narcissism	5.63	0.75	1	[0.14, 0.32]	[0.18, 0.37]	[−0.02, 0.18]	[0.19, 0.37]	[0.16, 0.35]	[0.25, 0.43]	[0.06, 0.26]
2. Collective self‐esteem	5.61	0.75	.23***	1	[0.09, 0.29]	[−0.42, −0.24]	[−0.09, 0.11]	[−0.06, 0.14]	[0.28, 0.45]	[0.52, 0.65]
3. Outgroup conspiracy belief	5.65	1.17	.28***	.19***	1	[−0.09, 0.11]	[0.47, 0.61]	[0.004, 0.20]	[0.18, 0.36]	[0.08, 0.28]
4. Ingroup conspiracy belief	4.33	0.94	.08	−.34***	.01	1	[0.02, 0.22]	[0.11, 0.30]	[−0.32, −0.13]	[−0.61, −0.47]
5. Perceived threat	5.22	1.48	.28***	.01	.54***	.12*	1	[0.17, 0.35]	[0.08, 0.27]	[−0.12, 0.08]
6. Instrumental treatment	4.63	0.87	.25***	.04	.10*	.21***	.26***	1	[0.02, 0.22]	[−0.08, 0.12]
7. Victim consciousness	5.27	1.12	.34***	.37***	.27***	−.23***	.17**	.12*	1	[0.34, 0.51]
8. System‐justifying belief	5.16	1.12	.16**	.59***	.18***	−.54***	−.02	.02	.43***	1

*Note*: Below the diagonal are Pearson correlation coefficients; above the diagonal are the corresponding 95% confidence intervals.

* *p* < .05;

** *p* < .01;

*** *p* < .001.

**FIGURE 2:**
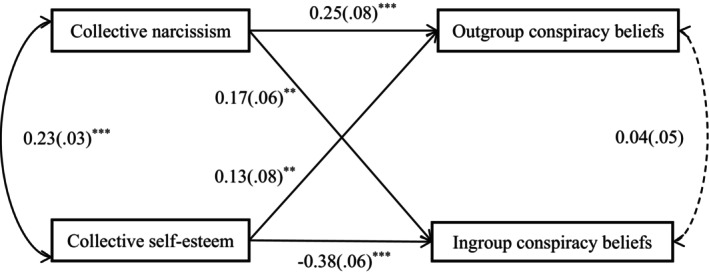
Effects of collective narcissism and collective self‐esteem on different types of conspiracy beliefs. **p < .01, ***p < .001. Entries are standardized regression coefficients with standard errors in parentheses. Dotted line indicates a non‐significant path.

As in Study 1, we used the *lavaan* package of *R* (Rosseel, 2012) to again test the path model. Results are displayed in Figure 2. As the model is again saturated, we focus our interpretation only on the individual paths. Collective narcissism was positively associated with outgroup conspiracy beliefs, *β* = .248, *SE* = .078, *p* < .001, 95% CI [0.235, 0.542], and ingroup conspiracy beliefs, *β* = .169, *SE* = .061, *p* = .001, 95% CI [0.093, 0.334]. Collective self‐esteem was positively associated with outgroup conspiracy beliefs, *β* = 0.133, *SE* = .078, *p* = .007, 95% CI [0.056, 0.362] but negatively associated with ingroup conspiracy beliefs, *β* = −.375, *SE* = .061, *p* < .001, 95% CI [−0.593, −0.353]. When gender and age were included as covariates, all the paths were still significant and in the predicted direction, supporting our hypotheses. These results are consistent with the findings in Study 1.

**FIGURE 3:**
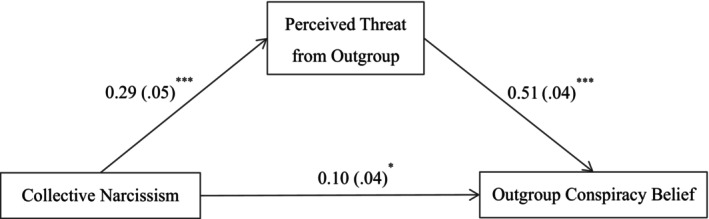
Mediation model. Path values are the path coefficients with standard errors. *p < .05, ***p < .001. All variables were standardized. Collective self‐esteem as a covariate. The use of bootstrapped estimators does not alter the path coefficients and results in only minor changes to the standard errors.

We then first tested if perceived outgroup threat would mediate the link between collective narcissism and outgroup conspiracy beliefs, after controlling for collective self‐esteem. The total effect of collective narcissism on outgroup conspiracy beliefs was significant (total effect = 0.25, 95% CI [0.15, 0.35]). As shown in Figure 3, collective narcissism was positively related to perceived threat from the outgroup (*B* = 0.29, *p* < .001, 95% CI [0.20, 0.39]); in turn, perceived threat from the outgroup was positively related to outgroup conspiracy beliefs (*B* = 0.51, *p* < .001, 95% CI [0.43, 0.60]). The residual direct effect was still significant (*B* = 0.10, *p* = .031, 95% CI [0.01, 0.19]). Perceived threat from the outgroup therefore partially mediated the link between collective narcissism and outgroup conspiracy beliefs (indirect effect = 0.15, 95% CI [0.10, 0.22]), and the proportion of the mediating effect was 60.76%. This supported Hypothesis 1.

**FIGURE 4:**
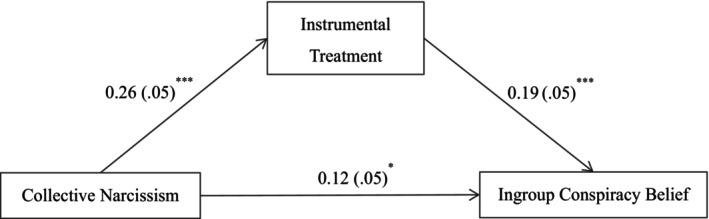
Mediation model. Path values are the path coefficients with standard errors. *p < .05, ***p < .001. All variables were standardized. Collective self‐esteem as a covariate. The use of bootstrapped estimators does not alter the path coefficients and results in only minor changes to the standard errors.

We then tested if instrumental treatment of ingroup members would mediate the effect of collective narcissism on ingroup conspiracy beliefs, after controlling for collective self‐esteem. The total effect of collective narcissism on ingroup conspiracy beliefs was significant (total effect = 0.17, 95% CI [0.07, 0.26]). As shown in Figure 4, collective narcissism was positively related to instrumental treatment of ingroup members (*B* = 0.26, *p* < .001, 95% CI [0.16, 0.36]); in turn, instrumental treatment of ingroup members was positively related to ingroup conspiracy beliefs (*B* = 0.19, *p* < .001, 95% CI [0.10, 0.29]). The residual direct effect was still significant (*B* = 0.12, *p* = .017, 95% CI [0.02, 0.22]). Instrumental treatment of ingroup members therefore partially mediated the link between collective narcissism and ingroup conspiracy beliefs (indirect effect = 0.05, 95% CI [0.02, 0.09]), and the proportion of the mediating effect was 29.45%. The results supported Hypothesis 2.

**FIGURE 5:**
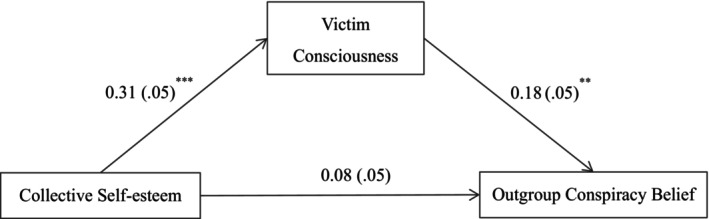
Mediation model. Path values are the path coefficients with standard errors. **p < .01, ***p < .001. All variables were standardized. Collective narcissism as a covariate. The use of bootstrapped estimators does not alter the path coefficients and results in only minor changes to the standard errors.

Next, we tested if victim consciousness would mediate the effect of collective self‐esteem on outgroup conspiracy beliefs, after controlling for collective narcissism. The results of the mediation analysis showed that the total effect of collective self‐esteem on outgroup conspiracy beliefs was significant (total effect = 0.13, 95% CI [0.04, 0.23]). As shown in Figure 5, collective self‐esteem was positively related to victim consciousness (*B* = 0.31, *p* < .001, 95% CI [0.21, 0.40]); in turn, victim consciousness was positively related to outgroup conspiracy beliefs (*B* = 0.18, *p* = .001, 95% CI [0.07, 0.28]). The residual direct effect was not significant (*B* = 0.08, *p* = .131, 95% CI [− 0.02, 0.18]). Victim consciousness therefore fully mediated the link between collective self‐esteem and outgroup conspiracy beliefs (indirect effect = 0.05, 95% CI [0.02, 0.10]), and the proportion of the mediating effect was 40.78%. The results supported Hypothesis 3a.

**FIGURE 6:**
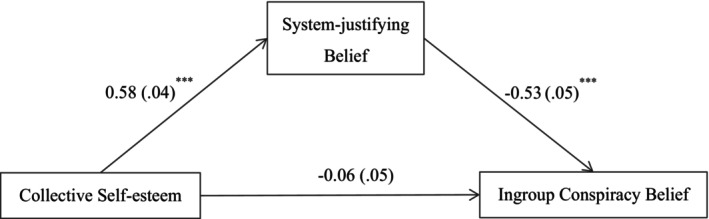
Mediation model. Path values are the path coefficients with standard errors. ***p < .001. All variables were standardized. Collective narcissism as a covariate. The use of bootstrapped estimators does not alter the path coefficients or the standard errors.

Finally, we tested if system‐justifying belief would mediate the effect of collective self‐esteem on ingroup conspiracy beliefs, after controlling for collective narcissism. The total effect of collective self‐esteem on ingroup conspiracy beliefs was significant (total effect = − 0.37, 95% CI [− 0.47, − 0.28]). As shown in Figure 6, collective self‐esteem was positively related to system‐justifying belief (*B* = 0.58, *p* < .001, 95% CI [0.50, 0.67]); in turn, system‐justifying belief was negatively related to ingroup conspiracy beliefs (*B* = − 0.53, *p* < .001, 95% CI [− 0.63, − 0.43]). The residual direct effect was not significant (*B* = − 0.06, *p* = .229, 95% CI [− 0.17, 0.04]). System‐justifying belief therefore fully mediated the link between collective self‐esteem and ingroup conspiracy beliefs (indirect effect = − 0.31, 95% CI [− 0.40, − 0.23]), and the proportion of the mediating effect was 82.98%. The results supported Hypothesis 4a.

### Discussion

Study 2 replicated the results of Study 1, suggesting that the relationships between collective narcissism, collective self‐esteem and two different conspiracy beliefs are robust in a Chinese context. More importantly, the results of Study 2 support Hypotheses 1, 2, 3a and 4a by revealing the underlying psychological mechanisms of these specific relationships in the Chinese context through four mediating processes.

## STUDY 3

Study 3 was the same questionnaire survey as Study 2 but was conducted in the United States. We expected to replicate the results of Studies 1 and 2 on the relationships between collective narcissism and two different conspiracy beliefs, thereby demonstrating consistency in these specific relationships. However, we expected to replicate the findings of previous studies in the United States on the relationships between collective self‐esteem and two different conspiracy beliefs (e.g. Biddlestone et al., 2022; Cichocka, Marchlewska, Golec de Zavala, & Olechowski, 2016). Therefore, we proposed Hypotheses 3b and 4b.

Hypothesis 3b In the United States, after controlling for collective narcissism, collective self‐esteem is negatively associated with outgroup conspiracy beliefs.

Hypothesis 4b In the United States, after controlling for collective narcissism, collective self‐esteem is negatively associated with ingroup conspiracy beliefs.

In Study 3, we tested Hypotheses 1, 2, 3b and 4b.^3^

### Method

#### Participants and design

407 American adult participants were recruited through Prolific. Ten participants were excluded because they failed the attention test (same as in Study 1). The final sample for data analysis consisted of 397 participants (198 male, 195 female, 4 other, *M*_age_ = 40.34, *SD* = 14.15).

### Materials and procedure

The 9‐item collective narcissism scale (*α* = .94), 16‐item collective self‐esteem scale (*α* = .88), 7‐item ingroup conspiracy beliefs scale (*α* = .81), 8‐item victim consciousness scale (*α* = .91) and 8‐item system‐justifying belief scale (*α* = .86) were measured as in Study 2. However, the collective, ingroup and social system referenced in these measures all pertain to the United States. The 7‐item outgroup (Chinese) conspiracy beliefs scale (*α* = .94) and 4‐item perceived outgroup threat scale (*α* = .91) were measured as in Study 2, but the outgroup referred to in these items was changed from the United States to China. In addition, instrumental treatment of ingroup members also used the same 7‐item scale (*α* = .72) as in Study 2, but participants were instructed to recall their own interactions or work with US government officials or government staff, as well as to evaluate their own relationships with them. Finally, we collected basic demographic information from all participants and gave them a small monetary reward.

### Results

#### Main analyses

**FIGURE 7:**
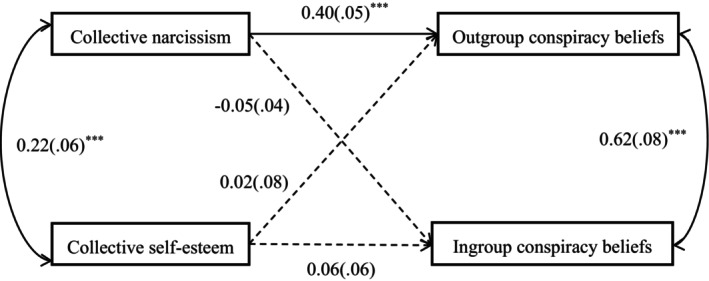
Effects of collective narcissism and collective self‐esteem on different types of conspiracy beliefs. ***p < .001. Entries are standardized regression coefficients with standard errors in parentheses. The dotted line indicates a non‐significant path.

Table 3 shows the results of descriptive statistics and correlational analyses for all variables. As in Studies 1 and 2, we again used the *lavaan* package of *R* (Rosseel, 2012) to test the path model to avoid the mutual influence caused by the overlap of collective narcissism and collective self‐esteem. Results are displayed in Figure 7. Collective narcissism was positively associated with outgroup conspiracy beliefs, *β* = .401, *SE* = 0.048, *p* < .001, 95% CI [0.307, 0.495], but not ingroup conspiracy beliefs, *β* = −.053, *SE* = 0.038, *p* = .161, 95% CI [−0.127, 0.021]. The effects of collective self‐esteem on outgroup and ingroup conspiracy beliefs were both non‐significant. For outgroup conspiracy beliefs, *β* = .015, *SE* = 0.081, *p* = .850, 95% CI [−0.143, 0.173]; for ingroup conspiracy beliefs, *β* = .063, *SE* = 0.063, *p* = .317, 95% CI [−0.061, 0.188]. When gender and age were included as covariates, the predictive results for all paths showed no significant changes.

**TABLE 3:** Descriptive analysis and correlations (Study 3).

	*M*	*SD*	1	2	3	4	5	6	7	8
1. Collective narcissism	3.13	1.44	1	[0.08, 0.27]	[0.31, 0.47]	[−0.16, 0.04]	[0.24, 0.41]	[−0.10, 0.10]	[0.60, 0.71]	[0.56, 0.68]
2. Collective self‐esteem	4.90	0.86	.18***	1	[−0.02, 0.18]	[−0.06, 0.14]	[0.03, 0.22]	[−0.02, 0.17]	[−0.05, 0.15]	[0.06, 0.25]
3. Outgroup conspiracy belief	4.07	1.47	.39***	.08	1	[0.29, 0.45]	[0.70, 0.78]	[0.01, 0.20]	[0.15, 0.33]	[−0.003, 0.19]
4. Ingroup conspiracy belief	4.90	1.07	−.06	.04	.37***	1	[0.13, 0.32]	[−0.05, 0.15]	[−0.24, −0.05]	[−0.50, −0.34]
5. Perceived threat	4.43	1.48	.33***	.13*	.74***	.23***	1	[0.01, 0.21]	[0.12, 0.31]	[0.04, 0.23]
6. Instrumental treatment	4.39	0.92	−.003	.08	.10*	.05	.11*	1	[−0.11, 0.09]	[−0.14, 0.06]
7. Victim consciousness	2.51	1.18	.66***	.05	.24***	−.15**	.22***	−.01	1	[0.35, 0.51]
8. System‐justifying belief	3.40	1.13	.62***	.15**	.10	−.42***	.14**	−.04	.43***	1

*Note*: Below the diagonal are Pearson correlation coefficients; above the diagonal are the corresponding 95% confidence intervals.

* *p* < .05;

** *p* < .01;

*** *p* < .001.

**FIGURE 8:**
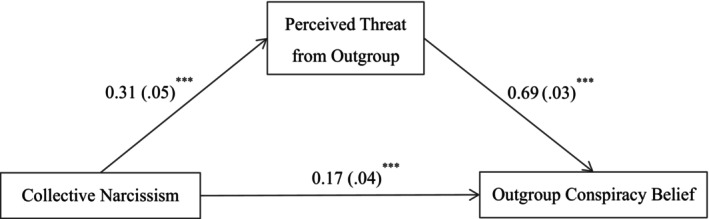
Mediation model. Path values are the path coefficients with standard errors. ***p < .001. All variables were standardized. Collective self‐esteem as a covariate. The use of bootstrapped estimators does not alter the path coefficients or the standard errors.

Given these results, we then only tested if perceived outgroup threat mediated the effect of collective narcissism on outgroup conspiracy beliefs, after controlling for collective self‐esteem. The results of the mediation analysis showed that the total effect of collective narcissism on outgroup conspiracy beliefs was significant (total effect = 0.39, 95% CI [0.30, 0.48]). As shown in Figure 8, collective narcissism was positively related to perceived threat from the outgroup (*B* = 0.31, *p* < .001, 95% CI [0.22, 0.41]); in turn, perceived threat from the outgroup was positively related to outgroup conspiracy beliefs (*B* = 0.69, *p* < .001, 95% CI [0.62, 0.76]). The residual direct effect was still significant (*B* = 0.17, *p* < .001, 95% CI [0.11, 0.24]). Perceived threat from the outgroup therefore partially mediated the link between collective narcissism and outgroup conspiracy beliefs (indirect effect = 0.22, 95% CI [0.15, 0.29]), and the proportion of the mediating effect was 55.49%. The results supported the mediating process specified in Hypothesis 1.

#### China–US empirical comparison

Since Studies 2 and 3 used the same measures, in a more exploratory fashion, we integrated both datasets and analysed whether country (China versus the US) significantly moderated the effects that appeared inconsistent in these nations. This includes three paths: collective narcissism influencing ingroup conspiracy beliefs, and collective self‐esteem influencing both ingroup and outgroup conspiracy beliefs. The path of collective narcissism influencing ingroup conspiracy beliefs was moderated by country (*B* = 0.17, *t* = 2.20, *p* = .028, 95% CI [0.02, 0.32]). Similarly, the path of collective self‐esteem influencing ingroup conspiracy beliefs was also moderated by country (*B* = − 0.47, *t* = −5.30, *p* < .001, 95% CI [− 0.64, − 0.30]). However, the path of collective self‐esteem influencing outgroup conspiracy beliefs was not (*B* = 0.19, *t* = 1.71, *p* = .088, 95% CI [− 0.03, 0.41]). These analyses suggest that the apparent discrepancies in the relationships of collective narcissism with ingroup conspiracy beliefs, and collective self‐esteem with ingroup conspiracy beliefs, meaningfully differed between the Chinese and American samples.

### Discussion

The results of Study 3 only supported Hypothesis 1, which is consistent with the findings of a previous meta‐analysis (Golec de Zavala et al., 2022), indicating that the relationship between collective narcissism and outgroup conspiracy beliefs is consistent in China and the US. Also, we found that this relationship is partially mediated by perceived outgroup threat. Study 3 did not find a meaningful relationship between collective narcissism and ingroup conspiracy beliefs in the US context, which is consistent with earlier findings by Cichocka, Marchlewska, and Golec de Zavala (2016); Cichocka, Marchlewska, Golec de Zavala, and Olechowski (2016) but inconsistent with findings by Biddlestone et al. (2022). We speculate that this may be due to the fact that Biddlestone et al. (2022) conducted their study during the Trump administration in the United States, which might have influenced US citizens' conspiratorial attitudes toward their own government, while our study and Cichocka, Marchlewska, and Golec de Zavala (2016); Cichocka, Marchlewska, Golec de Zavala, and Olechowski (2016) were conducted during the administration of Democratic leaders. Research participants on Prolific are more likely to be Democrat than Republican; moreover, the polarized societal context during the Trump presidency may have exacerbated the link between national narcissism and ingroup conspiracy beliefs (Biddlestone et al., 2022).

In addition, Study 3 also did not find any meaningful relationships between collective self‐esteem and ingroup or outgroup conspiracy beliefs. Although this is not consistent with the pattern of significance observed by Cichocka, Marchlewska, Golec de Zavala, and Olechowski (2016), the negative correlations between non‐narcissistic ingroup positivity (operationalized as group‐level self‐investment in their Study 3) and both ingroup conspiracy beliefs and outgroup conspiracy beliefs in their study were weak. This may imply that the relationships between collective self‐esteem and two different conspiracy beliefs, if they emerge, have a low effect size and are likely influenced by situational or cultural factors.

## GENERAL DISCUSSION

By distinguishing between two different forms of ingroup positivity and ingroup versus outgroup conspiracy theories, we conducted three cross‐sectional questionnaire studies to further investigate the relationships between collective narcissism, collective self‐esteem and different conspiracy beliefs in China and the United States, as well as their potential mediating mechanisms. We find that only the relationship between collective narcissism and outgroup conspiracy beliefs is consistent in China and the US, which is partially mediated by increased outgroup threat. Furthermore, in China, collective narcissism is positively related to ingroup conspiracy beliefs (partially mediated by increased instrumental treatment of ingroup members), collective self‐esteem is positively related to outgroup conspiracy beliefs (fully mediated by increased victim consciousness), but it is negatively related to ingroup conspiracy belief (fully mediated by increased system‐justifying belief). None of these three relationships are significant in the United States, however.

These findings meaningfully extend scientists' knowledge in the following ways. First, the current findings confirm the insight that different types of ingroup positivity (narcissistic versus non‐narcissistic) have different relationships with conspiracy beliefs (e.g. Biddlestone et al., 2022; Cichocka, Marchlewska, Golec de Zavala, & Olechowski, 2016; Golec de Zavala et al., 2022). Of these effects, however, only collective narcissism's impact on outgroup conspiracy beliefs turns out to be consistent in China and the US. This is consistent with the meta‐analytic result that collective narcissism is significantly positively associated with different types of conspiracy beliefs, but the relationship with outgroup conspiracy beliefs is the strongest (Golec de Zavala et al., 2022). Moreover, perceived outgroup threat was found to partially mediate the link between the two, which is consistent with the notion that collective narcissism predicts increased sensitivity to outgroup threats (Bertin et al., 2022; Cichocka, Marchlewska, & Biddlestone, 2022; Cichocka, Marchlewska, Golec de Zavala, & Olechowski, 2016; Golec de Zavala et al., 2022).

Second, exploratory analyses aggregating the Studies 2 and 3 data showed that cultural context (i.e. country) moderated the links between collective narcissism and ingroup conspiracy beliefs, and between collective self‐esteem and ingroup conspiracy beliefs. A possible explanation for this pattern of results might be that ingroup conspiracy beliefs specifically target the national government, and citizens' conspiratorial attitudes toward their own government are likely to vary by national background. However, country did not moderate the link between collective self‐esteem and outgroup conspiracy beliefs. We believe that the typical target of outgroup conspiracy beliefs varies across countries. For example, in China, such beliefs often focus on the United States, while in the United States, they may target China, Russia or other nations. As a result, some Americans may not view China as a typical outgroup conspiracy target, in the same way that Chinese individuals view the United States.

Third, by examining different mediating mechanisms, we have clarified the different motivational factors and psychological processes behind these specific relationships. In China, instrumental treatment of ingroup members partially mediates the link between collective narcissism and ingroup conspiracy beliefs. This is consistent with the findings of Biddlestone et al. (2022), showing the unique selfishness of collective narcissists, who put their own interests above those of the ingroup (Cichocka, Cislak, et al., 2022; Cislak et al., 2021; Golec de Zavala & Keenan, 2021; LaCroix & Pratto, 2015). This may also reflect their psychological process of projection (Douglas & Sutton, 2011): when individuals imagine themselves in power, they assume they would conspire against others, therefore projecting this mindset onto ingroup members—making them more likely to believe ingroup conspiracy theories.

Additionally, victim consciousness fully mediates the link between collective self‐esteem and outgroup conspiracy belief, while system‐justifying belief fully mediates the link between collective self‐esteem and ingroup conspiracy belief. These two effects were uniquely found in China. Compared to the United States, China has a distinct victim consciousness due to historical and ongoing external threats. Our findings show that even secure, non‐defensive ingroup positivity can trigger victim consciousness among Chinese citizens. Additionally, from a power dynamics perspective (Li et al., 2023), the power asymmetry between China and the United States leads the Chinese to perceive themselves as the victim in their intergroup interactions. Reflecting this asymmetry, Chinese conspiracy beliefs about the United States are stronger than Americans' beliefs about China (van Prooijen & Song, 2021).

Beyond the cultural uniqueness of victim consciousness, China's one‐party system creates a relatively strong link between the ingroup (own government and its agents) and the general social system (also highly associated with own government). Thus, ingroup conspiracy theories criticize what system‐justifying beliefs defend. By contrast, the US two‐party system means people's ingroup attitudes are influenced more strongly by political orientation, making them less likely to defend a government led by the opposing party. These dynamics may render the relationship between ingroup positivity and ingroup conspiracy beliefs more variable and complex in the United States.

Furthermore, our findings also challenge a key assumption that conspiracy beliefs are a homogeneous phenomenon, that different conspiracy beliefs share similar causes and consequences and that they support each other in a mutually reinforcing belief network (a ‘monological belief system’; Goertzel, 1994; Williams et al., 2022; see also Mao, Van Prooijen, & Van Lange, 2024). Our findings are consistent with the notion that ingroup versus outgroup conspiracy beliefs have different predictors. In addition, ingroup and outgroup conspiracy beliefs were positively correlated only in Study 3, but not in Studies 1 and 2, which is similar to earlier findings that ingroup and outgroup conspiracy beliefs cannot be simply regarded as the same psychological construct (Wang & Van Prooijen, 2023). Therefore, in future research, we also call on researchers to distinguish between different conceptualizations of conspiracy theories.

### Strengths, limitations and future directions

The current research has some noteworthy strengths. First, except for the first exploratory study, the other two studies were preregistered, and the results found in China were consistent, suggesting that our findings are robust. Second, by carrying out the research in the two cultural contexts of China and the United States, it not only suggests consistency of the relationship between collective narcissism and outgroup conspiracy beliefs, but also finds the uniqueness of the relationships between collective narcissism and ingroup conspiracy beliefs, and between collective self‐esteem and ingroup conspiracy beliefs. Finally, given that most previous research related to ingroup positivity and conspiracy theories has been conducted in the White, Educated, Industrialized, Rich and Democratic (WEIRD) samples (Biddlestone et al., 2021), the current research also extends the diversity of the samples to investigate these relationships.

However, there are some limitations in the current research that need to be addressed and explored in future studies. First, our data are cross‐sectional, which limits us to establish causal relationships between different forms of ingroup positivity and different conspiracy beliefs. Although some researchers have proposed that ingroup positivity (e.g. collective narcissism) is relatively stable over time, which is more likely to act as an antecedent of conspiracy beliefs than the other way around (Sternisko et al., 2023), more work is needed to provide evidence for this assumption. Second, since ingroup positivity and intergroup conspiracy theories in this study are framed within national or ethnic dimensions, this restricts us from extending our conclusions to broader group contexts, such as political parties or workplaces. Future research should further explore these diverse group dimensions.

In addition, there are some potential confounding factors that were not accounted for in this study, such as the political orientation of participants, and whether the Democrats or Republicans are in power in the United States. As US party affiliation adds complexity to these issues, future research may not only include measures of party affiliation but also classify and meta‐analyse the accumulating research evidence on this topic according to the period in which different parties were in power. Finally, de Golec Zavala et al. (2009) distinguished between two different types of collective narcissism based on the ‘agency‐communion model’ of individual narcissism. Communal collective narcissism, however, seems to better capture people's collective narcissism in the context of collectivist cultures. These different forms of collective narcissism in different cultural contexts provide opportunities for more fine‐grained future research.

## CONCLUDING REMARKS

Narcissistic (e.g. collective narcissism) and non‐narcissistic (e.g. collective self‐esteem) ingroup positivity are associated differently with conspiracy beliefs. Moreover, not all forms of ingroup positivity benefit ingroups. The current research suggests that collective narcissism may act as a ‘sweet poison’, fostering belief in ingroup conspiracy theories. This highlights the need to distinguish between narcissistic and non‐narcissistic ingroup positivity when assessing its social implications. Additionally, unlike outgroup conspiracy belief, the relationship between different forms of ingroup positivity and ingroup conspiracy belief varies across cultural contexts. These relationships may reflect cultural differences between China and the United States, which may be attributable to various political and contextual dynamics. Taken together, the current findings expand scientists' understanding of the link between ingroup positivity and conspiracy beliefs.

## AUTHOR CONTRIBUTIONS

**Jia‐Yan Mao:** Conceptualization; methodology; software; data curation; investigation; validation; formal analysis; supervision; funding acquisition; visualization; project administration; resources; writing – original draft; writing – review and editing. **Cai‐Yu Tian:** Conceptualization; methodology; software; visualization; formal analysis; writing – original draft. **Shen‐Long Yang:** Conceptualization; data curation; supervision; writing – review and editing; methodology; validation. **Jan‐Willem van Prooijen:** Conceptualization; methodology; supervision; visualization; project administration; resources; writing – review and editing; writing – original draft.

## FUNDING INFORMATION

This work was funded by China Scholarship Council (Grant/Award Number: 202006860004).

## CONFLICT OF INTEREST STATEMENT

No conflict of interest exists in the submission of this manuscript, and the manuscript has been approved by all authors before submission.

## ETHICS APPROVAL STATEMENT

All the studies were approved by the appropriate ethics review board. All ethical guidelines for human subjects' research were followed.

## Supporting information


Data S1.

## Data Availability

All data and materials of the studies reported here are publicly available on the Open Science Framework (https://osf.io/a69se/files/osfstorage).

## Footnotes

^1^
The questionnaire also included measures of other variables, such as collective action, perceived control, etc., which served other research projects.

^2^
Please refer to the online Supporting Information for the Chinese versions of the scales and their sources.

^3^
To remain consistent with the measures in Study 2, we included victim consciousness and system‐justifying belief in Study 3 as well. However, as described in Hypotheses 3b and 4b, we did not expect these two variables to show the same mediating effects in the US as they did in China.
