# Supplementary material for: ‘Sweet poison’ and ‘mild medicine’: Different effects of collective narcissism and collective self‐esteem on ingroup versus outgroup conspiracy beliefs
Source: Br J Psychol. 2025 Sep 26;117(1):356–77. doi: 10.1111/bjop.70032 (PMC12783876; doi:10.1111/bjop.70032)
Supplement: Supplementary file 1 — Data S1. [file BJOP-117-356-s001.docx]

**Supplemental Material 1**

Complete measuring materials in Study 1:

Measurement of collective narcissism (5 items, 1 = *strongly disagree*, 7 = *strongly agree*):

***English Version***

If China had a major say in the world, the world would be a much better place.

China deserves special treatment.

It really makes me angry when others criticize China.

Not many people seem to fully understand the importance of China.

I will never be satisfied until China gets all it deserves.

***Chinese Version***

如果中国在世界上拥有重要的发言权，世界将会变得更加美好。

中国值得特别对待。

当别人批评中国时，我会感到生气。

似乎没有多少人完全理解中国的重要性。

在中国得到应有的认可之前，我永远不会满意。

The Chinese version of the scale is referenced from:

Xu, B. (2020). *How does system threat affect collective narcissism? an integral view of motivation and cognition* (Doctoral dissertation, Central China Normal University). [In Chinese]. https://doi.org/10.27159/d.cnki.ghzsu.2020.003967

Measurement of collective self-esteem (16 items, 1 = *strongly disagree*, 7 = *strongly agree*):

***English Version***

**Based on your current feelings as a member of the Chinese community**, please select the score that best reflects your agreement with each of the following statements.

I am a worthy member of the social groups I belong to.

I often regret that I belong to the social groups I do. (R)

Overall, my social groups are considered good by others.

Overall, my group memberships have very little to do with how I feel about myself. (R)

I feel I don't have much to offer to the social groups I belong to. (R)

In general, I'm glad to be a member of the social groups I belong to.

Most people consider my social groups, on the average, to be more ineffective than other social groups. (R)

The social groups I belong to are an important reflection of who I am.

I am a cooperative participant in the social groups I belong to.

Overall, I often feel that the social groups of which I am a member are not worthwhile. (R)

In general, others respect the social groups that I am a member of.

The social groups I belong to are unimportant to my sense of what kind of a person I am. (R)

I often feel I'm a useless member of my social groups. (R)

I feel good about the social groups I belong to.

In general, others think that the social groups I am a member of are unworthy. (R)

In general, belonging to social groups is an important part of my self-image.

***Chinese Version***

**根据你现在作为中国人这个集体里一员的感受**，请选择一个最接近您感觉的分数，说明您对下面每项描述的赞成程度如何。

我是这个集体中一名有价值的成员。

我经常后悔自己属于这个集体。

总的来说，其他国家的人认为我们这个集体是好的。

总的来说，这个集体对于自己的影响很小。

我总觉得我对这个集体没有多大贡献。

一般来说，我对自己是这个集体的一员感到高兴。

多数人认为我们这个集体总的来说比其他集体（外国人）的效率要差。

我从属的这个集体是我个人身份的重要标志。

我以合作态度参与这个集体。

总的来说，我经常感到这个集体没有什么存在价值。

一般来说，其他人尊重我们集体。

这个集体对于我感受自己是怎样一个人并不重要。

我经常觉得自己是集体里一名没有用处的成员。

我对我从属的这个集体感觉良好。

一般来说，其他集体的人（外国人）认为我们这个集体没有什么价值。

一般来说，对集体成员的身份是我自我形象的重要部分。

The Chinese version of the scale is referenced from:

Lü, X., Zhong, N., Tian, B., & Shu, S. (2017). The Influence of Different Types of Intergroup Threat on Collective Self-esteem: The mediating effect of two kinds of emotions. *Journal of Psychological Science, 40*(3), 632–637. [In Chinese]. https://doi.org/10.16719/j.cnki.1671-6981.20170319

Measurement of American (Outgroup) conspiracy beliefs (7 items, 1 = *strongly disagree*, 7 = *strongly agree*):

***English Version***

The secret agency of the U.S. has been trying to influence political decision-making in China.

Companies in America are trying to take over China's economy.

The American government is secretly conspiring to harm China.

American secret agencies are trying to use genetically modified organisms as a bio-terrorism weapon to kill Chinese.

American secret agencies often try to change the people who are in power in China.

The American government is attempting to interfere with the Chinese government and control China.

The American government is trying to split China from its allies.

***Chinese Version***

美国的秘密机构一直试图影响中国的政治决策。

美国的公司试图控制中国的经济。

美国政府暗中阴谋伤害中国。

美国秘密机构试图利用转基因食品作为杀死中国人的生物恐怖主义武器。

美国的秘密机构正试图改变在中国掌权的人。

美国政府试图干涉中国政府并控制中国。

美国政府正试图将中国从盟国分裂出去。

The Chinese version of the scale is referenced from:

Van Prooijen, J. W., & Song, M. (2021). The cultural dimension of intergroup conspiracy theories. *British Journal of Psychology, 112*(2), 455–473. https://doi.org/10.1111/bjop.12471

Measurement of ingroup conspiracy beliefs (6 items, 1 = *strongly disagree*, 7 = *strongly agree*):

***English Version***

Those at the top do whatever they want.

Those at the top with power determine the destiny of millions.

I think that the various conspiracy theories about those at the top circulating in the media are absolute nonsense. (R)

There are many secret decisions made by those at the top that are not open to us.

There is no good reason to distrust those at the top and the official media. (R)

Most people do not recognize to what extent our life is determined by conspiracies secretly orchestrated by those at the top.

***Chinese Version***

那些在上层的人可以做他们想做的任何事情。

拥有权力的上层可以决定数百万人的命运。

我认为媒体中传播的关于上层的各种阴谋论绝对是胡说八道。

上层有很多秘密决策并不对我们公开。

并没有什么好的理由去不相信上层和官方媒体。

大部分人不明白我们的生活有多少是被上层秘密策划的阴谋所决定的。

The Chinese version of the scale is referenced from:

Mao, J. Y. (2020). *The influence of social class on conspiracy beliefs: The roles of sense of control, need for structure, and regulatory focus* (Master’s thesis, Nanjing Normal University). [In Chinese]. https://doi.org/10.27245/d.cnki.gnjsu.2020.002212

**Supplemental Material 2**

Complete other measuring materials in Study 2:

Measurement of collective narcissism (9 items, 1 = *strongly disagree*, 7 = *strongly agree*):

***English Version***

I wish other groups would more quickly recognize the authority of China.

China deserves special treatment.

I will never be satisfied until China gets the recognition it deserves.

I insist upon China getting the respect that is due to it.

It really makes me angry when others criticize China.

If China had a major say in the world, the world would be a much better place.

I do not get upset when people do not notice achievements of China. (R)

Not many people seem to fully understand the importance of China.

The true worth of China is often misunderstood.

***Chinese Version***

我希望别的国家能尽早认识到中国在国际上的权威地位。

中国在国际上应该享有特别的待遇。

如果中国没有得到应有的认可，我是不会满足的。

我坚持中国要得到它所应得的尊重。

别的国家对中国的批评会令我愤愤不平。

假如中国能在世界上起主导作用，世界将会好很多。

即便世人注意不到中国的成就，我也不会为此感到不快。

充分理解中国的重要性的人似乎还不多。

中国的真正价值经常被误解。

The Chinese version of the scale is referenced from:

Xu, B. (2020). *How does system threat affect collective narcissism? an integral view of motivation and cognition* (Doctoral dissertation, Central China Normal University). [In Chinese]. https://doi.org/10.27159/d.cnki.ghzsu.2020.003967

Measurement of ingroup conspiracy beliefs (7 items, 1 = *strongly disagree*, 7 = *strongly agree*):

***English Version***

Those at the top do whatever they want.

Those at the top with power determine the destiny of millions.

I think that the various conspiracy theories about those at the top circulating in the media are absolute nonsense. (R)

There are many secret decisions made by those at the top that are not open to us.

Whenever there is a major social event, those at the top will not hide information from the public. (R)

There is no good reason to distrust those at the top and the official media. (R)

Most people do not recognize to what extent our life is determined by conspiracies secretly orchestrated by those at the top.

***Chinese Version***

那些在上层的人可以做他们想做的任何事情。

拥有权力的上层可以决定数百万人的命运。

我认为媒体中传播的关于上层的各种阴谋论绝对是胡说八道。

上层有很多秘密决策并不对我们公开。

每当发生重大的社会事件，上层不会对公众隐瞒信息。

并没有什么好的理由去不相信上层和官方媒体。

大部分人不明白我们的生活有多少是被上层秘密策划的阴谋所决定的。

The Chinese version of the scale is referenced from:

Mao, J. Y. (2020). *The influence of social class on conspiracy beliefs: The roles of sense of control, need for structure, and regulatory focus* (Master’s thesis, Nanjing Normal University). [In Chinese]. https://doi.org/10.27245/d.cnki.gnjsu.2020.002212

Measurement of instrumental treatment of ingroup members (7 items, 1 = *strongly disagree*, 7 = *strongly agree*):

***English Version***

I think more about what they can do for me than what I can do for them.

I tend to contact them only when I need something from them.

I try to motivate them to do things that will help me succeed.

The relationship is important to me because it helps me accomplish my goals.

They are very useful to me.

If the nature of my job (or their jobs) changed and they were not helpful anymore, the relationship probably wouldn't continue.

Someone else who have the same role could become equally important to me.

***Chinese Version***

我更多地考虑他们能为我做什么，而不是我能为他们做什么。

我倾向于只在我需要他们时才联系他们。

我试图激励他们去做一些对我成功有帮助的事情。

这段关系对我很重要，因为它有助于我实现我的目标。

他们对我非常有用。

如果我的工作性质（或他们的工作性质）发生变化，而他们对我不再有帮助，我们的关系可能不会继续。

具有相同作用的其他人对我可能同样重要。

Measurement of perceived threat from America (4 items, 1 = *strongly disagree*, 7 = *strongly agree*):

***English Version***

America's international status is a critical threat to China.

America's nuclear weapons are a critical threat to China.

America's military power is a critical threat to China.

America's technological prowess is a critical threat to China.

***Chinese Version***

美国的国际地位是对中国的重大威胁。

美国的核武器是对中国的重大威胁。

美国的军事实力是对中国的重大威胁。

美国的科技实力是对中国的重大威胁。

Measurement of victim consciousness (8 items, 1 = *strongly disagree*, 7 = *strongly agree*):

***English Version***

While other countries around the world have been harmed in the past and the present, China's experience is overall much more severe.

No other country went through similar hardships as China.

While all experiences of suffering are somewhat different, the Chinese experience is truly unique.

It is simply wrong to say that other countries went through the same amount of hardships as the Chinese people.

No country has suffered as much as the Chinese people.

There is no suffering of other countries that is comparable to China's suffering.

The harm the Chinese people suffered is unique in history.

Saying that other countries' experiences of suffering are similar to China's (experiences) is just wrong.

***Chinese Version***

虽然世界上其他国家在过去和现在都受到过伤害，但总体而言，中国经历的要严重得多。

没有其他国家经历过像中国这样的苦难。

虽然所有的苦难经历都有所不同，但中国的经历确实是独一无二的。

说其他国家和中国人民经历了同样多的苦难，这是完全错误的。

没有哪个国家遭受过中国人民那样多的苦难。

其他国家所遭受的苦难无法与中国相比。

中国人所遭受的伤害在历史上是独一无二的。

说其他国家的苦难经历与中国的相似是错误的。

None of the three scales—instrumental treatment of ingroup members, perceived threat from America, and victim consciousness—had existing Chinese versions. We translated all three scales into Chinese for the first time. The translation procedure was as follows: First, three psychology researchers fluent in both English and Chinese (including PhD students and an associate professor) independently translated the original English items into Chinese. Then, a Chinese native English major teacher back-translated the items into English. After multiple rounds of discussion and revision, the final Chinese versions were determined.

Measurement of system-justifying belief (8 items, 1 = *strongly disagree*, 7 = *strongly agree*):

***English Version***

In general, I find Chinese society to be fair.

In general, China's political system operates as it should.

Chinese society needs to be radically restructured.

China is the best country in the world to live in.

Most policies of the Chinese government serve the greater good.

Every Chinese has a fair shot at wealth and happiness.

Chinese society is getting worse every year.

Chinese society is set up so that people usually get what they deserve.

***Chinese Version***

中国社会是公平的。

中国政治体系的运作是公正的。

中国社会需要彻底地重建。

中国是世界上最好的国家之一。

中国政府的大多数政策都给民众带来了好处。

在追求财富和幸福时，每个中国人都有公平的机会。

中国社会正在变得越来越糟糕。

中国的社会体系能够保证每个人得到他所应得的。

The Chinese version of the scale is referenced from:

Yang, S. L. (2017). *The mechanism between social class and system-justifying belief: explorations based on attributional and compensatory perspectives* (Doctoral dissertation, Central China Normal University). [In Chinese]. https://doi.org/CNKI:CDMD:1.1017.266549

**Supplemental Material 3**

The following shows the complete mediation models in Study 2, with collective narcissism as the independent variable, collective self-esteem as a covariate, and ingroup/outgroup conspiracy beliefs as dependent variables.

Collective Self-esteem

Outgroup Conspiracy Belief

Perceived Threat from Outgroup

0.29 (.05)^***^

0.51 (.04)^***^

0.10 (.04)^*^

Collective Narcissism

-0.06 (.05)

0.16 (.04)^***^

Figure S1. The complete mediation model. Path values are the path coefficients with standard errors.

^*^*p* < 0.05, ^**^*p* < 0.01, ^***^*p* < 0.001. All variables were standardized. Collective narcissism as the independent variable. Collective self-esteem as a covariate.

Collective Self-esteem

Ingroup Conspiracy Belief

Instrumental Treatment

0.26 (.05)^***^

0.19 (.05)^***^

0.12 (.05)^*^

Collective Narcissism

-0.02 (.05)

-0.37 (.05)^***^

Figure S2. The complete mediation model. Path values are the path coefficients with standard errors.

^*^*p* < 0.05, ^**^*p* < 0.01, ^***^*p* < 0.001. All variables were standardized. Collective narcissism as the independent variable. Collective self-esteem as a covariate.

The following shows the complete mediation models in Study 2, with collective self-esteem as the independent variable, collective narcissism as a covariate, and ingroup/outgroup conspiracy beliefs as dependent variables.

Collective Narcissism

Outgroup Conspiracy Belief

Victim Consciousness

0.31 (.05)^***^

0.18 (.05)^**^

0.08 (.05)

Collective Self-esteem

0.27 (.05)^***^

0.20 (.05)^***^

Figure S3. The complete mediation model. Path values are the path coefficients with standard errors.

^*^*p* < 0.05, ^**^*p* < 0.01, ^***^*p* < 0.001. All variables were standardized. Collective self-esteem as the independent variable. Collective narcissism as a covariate.

Collective Narcissism

Ingroup Conspiracy Belief

System-justifying Belief

0.58 (.04)^***^

-0.53 (.05)^***^

-0.06 (.05)

Collective Self-esteem

0.02 (.04)

0.18 (.04)^***^

Figure S4. The complete mediation model. Path values are the path coefficients with standard errors.

^*^*p* < 0.05, ^**^*p* < 0.01, ^***^*p* < 0.001. All variables were standardized. Collective self-esteem as the independent variable. Collective narcissism as a covariate.

**Supplemental Material 4**

The following shows the complete mediation model in Study 3, with collective narcissism as the independent variable, collective self-esteem as a covariate, and outgroup conspiracy beliefs as the dependent variable.

Collective Self-esteem

Outgroup Conspiracy Belief

Perceived Threat from Outgroup

0.31 (.05)^***^

0.69 (.03)^***^

0.17 (.04)^***^

Collective Narcissism

0.07 (.05)

-0.04 (.03)

Figure S5. The complete mediation model. Path values are the path coefficients with standard errors.

^*^*p* < 0.05, ^**^*p* < 0.01, ^***^*p* < 0.001. All variables were standardized. Collective narcissism as the independent variable. Collective self-esteem as a covariate.
